# Functionally Overlapping Variants Control Tuberculosis Susceptibility in Collaborative Cross Mice

**DOI:** 10.1128/mBio.02791-19

**Published:** 2019-11-26

**Authors:** Clare M. Smith, Megan K. Proulx, Rocky Lai, Michael C. Kiritsy, Timothy A. Bell, Pablo Hock, Fernando Pardo-Manuel de Villena, Martin T. Ferris, Richard E. Baker, Samuel M. Behar, Christopher M. Sassetti

**Affiliations:** a Department of Microbiology and Physiological Systems, University of Massachusetts Medical School, Worcester, Massachusetts, USA; b Department of Genetics, University of North Carolina at Chapel Hill, Chapel Hill, North Carolina, USA; c Lineberger Comprehensive Cancer Center, University of North Carolina at Chapel Hill, Chapel Hill, North Carolina, USA; UC Berkeley

**Keywords:** *Mycobacterium tuberculosis*, adaptive immunity, Collaborative Cross mice, host genetics, host response, host-pathogen interactions

## Abstract

Host genetics plays an important role in determining the outcome of Mycobacterium tuberculosis infection. We previously found that Collaborative Cross (CC) mouse strains differ in their susceptibility to M. tuberculosis and that the CC042/GeniUnc (CC042) strain suffered from a rapidly progressive disease and failed to produce the protective cytokine gamma interferon (IFN-γ) in the lung. Here, we used parallel genetic and immunological approaches to investigate the basis of CC042 mouse susceptibility. Using a population derived from a CC001/Unc (CC001) × CC042 intercross, we mapped four quantitative trait loci (QTL) underlying tuberculosis immunophenotypes (*Tip1* to *Tip4*). These included QTL that were associated with bacterial burden, IFN-γ production following infection, and an IFN-γ-independent mechanism of bacterial control. Further immunological characterization revealed that CC042 animals recruited relatively few antigen-specific T cells to the lung and that these T cells failed to express the integrin alpha L (αL; i.e., CD11a), which contributes to T cell activation and migration. These defects could be explained by a CC042 private variant in the *Itgal* gene, which encodes CD11a and is found within the *Tip2* interval. This 15-bp deletion leads to aberrant mRNA splicing and is predicted to result in a truncated protein product. The *Itgal^CC042^* genotype was associated with all measured disease traits, indicating that this variant is a major determinant of susceptibility in CC042 mice. The combined effect of functionally distinct *Tip* variants likely explains the profound susceptibility of CC042 mice and highlights the multigenic nature of tuberculosis control in the Collaborative Cross.

## Abstract (precis)

The variable outcome of Mycobacterium tuberculosis infection observed in natural populations is difficult to model in genetically homogeneous small-animal models. The newly developed Collaborative Cross (CC) represents a reproducible panel of genetically diverse mice that display a broad range of phenotypic responses to infection. We explored the genetic basis of this variation, focusing on a CC line that is highly susceptible to M. tuberculosis infection. This study identified multiple quantitative trait loci associated with bacterial control and cytokine production, including one that is caused by a novel loss-of-function mutation in the *Itgal* gene, which is necessary for T cell recruitment to the infected lung. These studies verify the multigenic control of mycobacterial disease in the CC panel, identify genetic loci controlling diverse aspects of pathogenesis, and highlight the utility of the CC resource.

## INTRODUCTION

Nearly one-quarter of the world’s population has been exposed to Mycobacterium tuberculosis, yet less than 10% of these exposures progress to clinical disease (1). The rational design of more effective interventions requires an increased understanding of the factors that determine the outcome of this interaction. A large body of evidence supports an important role for host genetics in determining disease progression, including classic twin studies (2, 3), linkage analyses (4–8), and both case-control (9, 10) and genome-wide association (11, 12) studies. However, the genetic variants that determine the risk of adult pulmonary disease remain elusive due to both the complexity of factors influencing clinical outcomes and the lack of model systems that reflect the diversity of natural populations.

Much of the mechanistic insight into protective immunity against M. tuberculosis comes from mouse models of infection. Resistant strains of mice, such as the commonly used C57BL/6J (B6) strain, are able to restrict the replication of M. tuberculosis for over a year (13). Protective immunity in B6 mice relies heavily on Th1-biased CD4^+^ T cell activation and the production of gamma interferon (IFN-γ) in the infected tissue (14, 15). IFN-γ mediates its protective effect both by activating microbiocidal mechanisms in parasitized macrophages (16–18) and by inhibiting the recruitment of granulocytes that have been shown to exacerbate disease (19, 20). As these effects require the local production of the cytokine, the adhesion molecules and chemokines required for T cell recruitment play a pivotal role in immunity. Studies in knockout mice have shown that T cell expression of the integrin αLβ2 and the chemokine receptors CXCR3, CCR5, and CCR2 is important for the proper positioning of these T cells and for protective immunity in the lung (21–24).

Despite the wealth of mechanistic data that can be obtained in the mouse model, standard lab strains of mice do not reproduce the diversity in pathogenesis observed in natural populations. Not only does the relatively homogeneous histopathology observed in these animals differ from the variable disease seen in patients (25), but recent evidence suggests an unappreciated diversity in human immune responses to M. tuberculosis (26), which have not been described in mice. For example, it now appears that some humans have the capacity to control M. tuberculosis infection in the absence of the IFN-γ response that is critical in B6 mice (27), and emerging evidence also suggests a possible protective role for antibodies that play little role in the standard mouse model (28, 29). Much of the previous work to increase the diversity of tuberculosis (TB) disease in mice has focused on relatively susceptible substrains that remain closely related to B6 mice (30). While studies contrasting these strains have identified a number of quantitative trait loci (QTL) associated with susceptibility (31–36), the diversity observed in these highly related strains still does not mimic the diversity observed in an outbred population.

Recently, a number of new resources have become available to introduce additional genetic variability into mouse model systems. For example, Diversity Outbred (DO) mice are an outbred population of genetic mosaics based on eight inbred founders, including highly divergent wild-derived strains (37). M. tuberculosis infection of DO mice produces a wide range of disease manifestations, including extreme susceptibility, which is not observed in more standard lab strains (38). While the DO population incorporates a great deal of diversity, each genotype is represented by a single unique mouse, limiting the mechanistic characterization that is otherwise a strength of the mouse system. The Collaborative Cross (CC) consists of recombinant inbred lines derived from the same eight inbred founder strains on which the DO population is based (39). In contrast to the outbred DO population, each inbred CC strain represents a reproducible mosaic of the founder genomes and, thus, a reproducible model of disease (40). We have shown that the range of TB susceptibility observed in the DO population can be recapitulated in CC mice (41). This previous work found that CC042/GeniUnc (CC042) animals were highly susceptible to M. tuberculosis infection, in contrast to resistant mouse strains, such as CC001/Unc (CC001) or B6 mice (41).

Here we investigated the basis of TB susceptibility in the CC042 strain using a parallel genetic and immunophenotyping approach. By producing an intercross population based on CC042 and the resistant CC001 strains, we identified multiple QTL, named tuberculosis immunophenotypes (*Tip1* to *Tip4*), that were differentially associated with the bacterial burden and/or IFN-γ production. In this population, canonical IFN-γ-dependent immunity was controlled by a novel mutation in the *Itgal* gene, which disrupts expression of the αLβ2 adhesion molecule and prevents the recruitment of cytokine-expressing T cells to the site of infection. Other *Tip* loci were driven by wild-derived founder alleles that either reduce IFN-γ production or control IFN-γ-independent immunity. Together, these observations explain the extreme susceptibility of CC042 mice and indicate that the CC panel can be used to understand diverse mechanisms of protective immunity to M. tuberculosis.

## RESULTS

### Control of M. tuberculosis infection in CC042 mice is lost upon the onset of adaptive immunity.

**FIG 1:**
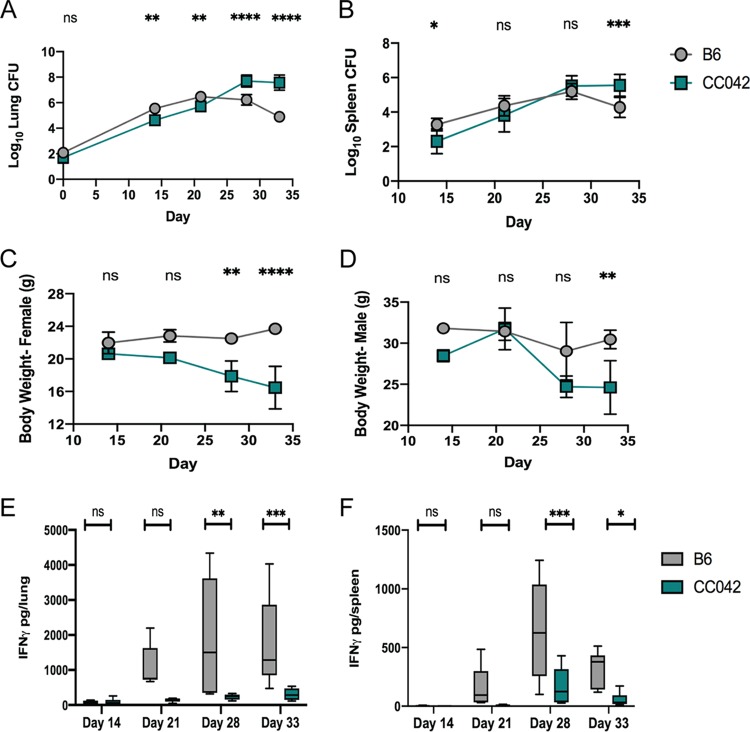
CC042 mice are susceptible to low-dose aerosol M. tuberculosis infection. The numbers of lung CFU (A), the numbers of spleen CFU (B), body weight (C, D), and total IFN-γ levels in lung (E) or spleen (F) homogenates at 14, 21, 28, and 33 days after infection by low-dose aerosol (∼50 to 100 CFU) of M. tuberculosis strain H37Rv are shown. All mice were infected in one batch, and 3 males and 3 females of each strain were used for analysis at each time point. The data in the graphs represent the mean ± SD. One-way analysis of variance with Sidak’s multiple-comparison test was used to determine significance. *, *P* < 0.05; **, *P* < 0.01; ***, *P* < 0.001; ****, *P* < 0.0001; ns, not significant.

In order to dissect the mechanisms underlying CC042 susceptibility to M. tuberculosis, we first profiled disease progression in aerosol-infected CC042 animals compared to the more resistant B6 mice. In the standard B6 mouse model, the peak of bacterial burden was observed at about 21 days postinfection, coincident with the onset of robust Th1 immunity. While CC042 mice had 10-fold lower numbers of CFU in the lung and spleen at 14 days than B6 mice (Fig. 1A and B), CC042 animals ultimately failed to control bacterial replication. By day 28 postinfection, the lungs of CC042 mice harbored 100-fold more bacteria than the lungs of B6 mice. Similar trends in relative bacterial burden were observed in the spleen. All CC042 mice had lost significant weight and required euthanasia because of morbidity by day 33 postinfection (Fig. 1C and D). Both male and female CC042 mice were similarly moribund at this time point.

**FIG 2:**
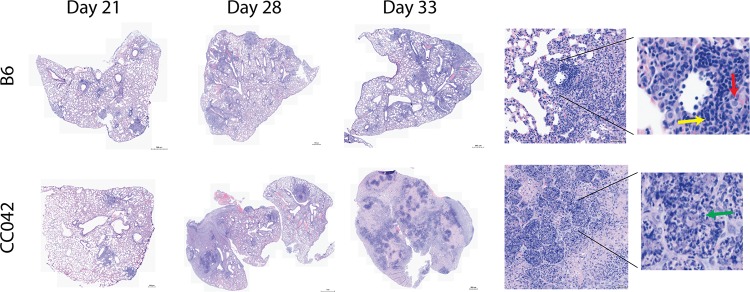
Changes in lung pathology during M. tuberculosis infection. Lung lobes obtained from B6 and CC042 mice at days 21, 28, and 33 postinfection were stained with hematoxylin and eosin. Images are representative of those from 6 mice per strain per time point. (Insets) Magnified images from day 33 show lymphocytes (yellow arrow), macrophages (red arrow), and neutrophils (green arrow).

The superior control of bacterial replication by B6 mice correlated with IFN-γ abundance in lung homogenate. There was a significant increase in IFN-γ levels starting on day 21, which peaked by day 28 in all B6 mice (Fig. 1E). In contrast, the concentration of IFN-γ in the lungs and spleens of CC042 mice remained relatively low throughout the course of infection (Fig. 1E and F). Further histological comparison found cellular infiltration in B6 mouse lung lesions that largely consisted of macrophages and lymphocytes throughout the experiment, while necrosis and neutrophil infiltration were apparent in CC042 mouse lungs by 21 days and were sustained until the termination of the experiment (day 33 postinfection) (Fig. 2). Together, these data suggest that the susceptibility of CC042 mice could be related to a defect in IFN-γ production that promotes bacterial growth and granulocyte infiltration.

### Identifying TB susceptibility loci in a CC001 × CC042 intercross.

To investigate the genetic basis of CC042 mouse susceptibility, we created an F_2_ population between CC042 and CC001 mice. The CC001 strain was chosen as a partner to cross with the CC042 strain because of its relative TB resistance (which is similar to that of B6 mice) (41) and to match the CC042 mouse major histocompatibility locus (H-2^b^). We crossed female CC001 mice with male CC042 mice to generate F_1_ progeny [(CC001 × CC042)F_1_ mice], which were then intercrossed to produce 201 F_2_ offspring. We infected male and female F_1_ and F_2_ progeny, along with parental strains, with M. tuberculosis (H37Rv) via low-dose aerosol. The mice were sacrificed at between 28 and 31 days postinfection, a time point that maximized phenotypic differences while minimizing morbidity. The phenotypes measured included the numbers of lung CFU, the numbers of spleen CFU, and lung IFN-γ levels.

**FIG 3:**
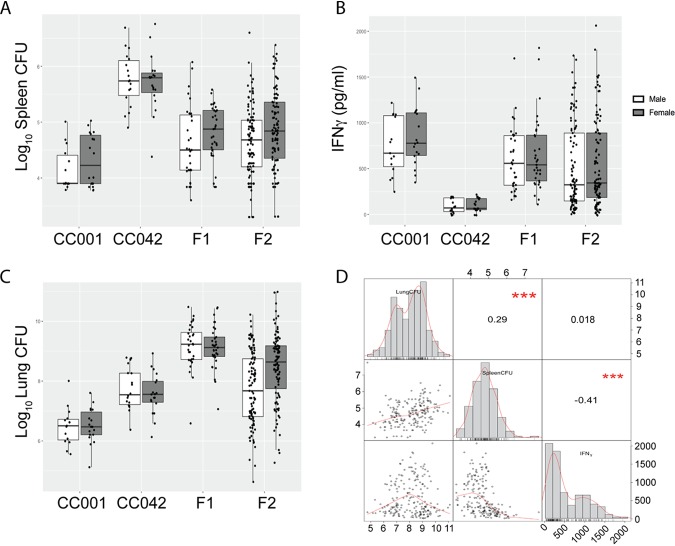
TB disease traits in a (CC001 × CC042)F_2_ intercross population. At 28 to 31 days postinfection, the following traits were quantified in the parental strains and the F_1_ and F_2_ offspring: the numbers of spleen CFU (A), IFN-γ levels from lung homogenate (B), and the numbers of lung CFU (C). (D) The Pearson correlation between measured traits. The distribution of each measured phenotype (numbers of lung CFU, numbers of spleen CFU, and IFN-γ levels) is shown on the diagonal. Scatter plots depicting the correlation for each pair of phenotypes are shown below the diagonal. Above the diagonal, the correlation coefficient and significance are shown. ***, *P* < 0.001. The data shown in all panels are for the following population sizes: for the F_2_ population, *n* = 201 total mice, *n* = 101 females, and *n* = 100 males; for the F_1_ population, *n* = 65 total mice, *n* = 32 females, and *n* = 33 males; for CC001 parent mice, *n* = 33 total mice, *n* = 15 females, and *n* = 18 males; and for CC042 parent mice, *n* = 37 total mice, *n* = 18 females, and *n* = 19 males. The mice were infected in 4 batches, and values were adjusted for batch differences using coefficients from multiple regressions.

For the numbers of spleen CFU and lung IFN-γ levels, F_1_ mice showed an intermediate phenotype and F_2_ mice displayed a distribution of values spanning the range for the parental strains (Fig. 3A and B). In contrast, F_1_ mice had higher bacterial burdens in the lung than the susceptible CC042 mice (Fig. 3C), and F_2_ mice spanned this greater phenotypic range. The three traits covaried in a predictable manner (Fig. 3D). The bacterial burden in lung and spleen were positively correlated. Lung IFN-γ levels were more strongly associated with the numbers of CFU in the spleen than with the numbers of CFU in the lung, consistent with the more prominent role of IFN-γ-independent T cell functions in the lung (42). The imperfect correlation between these traits, as well as the expansion of phenotypic ranges in F_2_ animals, suggested that multiple genes controlled the differences between CC001 and CC042 mice.

In total, 170 F_2_ mice (86 female and 84 male mice) were genotyped with the MiniMUGA array (43). We first validated our genetic mapping protocol using a coat color trait (see Fig. S1 in the supplemental material). CC042 mice have a white head spot (blaze) on their forehead, a characteristic inherited from the WSB/EiJ (designated for Watkins star blaze [WSB]) founder strain. One in every four F_2_ progeny carried a blaze, confirming autosomal recessive inheritance (44). The blaze and base coat color (black from CC001 mice or agouti from CC042 mice) assorted independently in the F_2_ offspring, as shown by the expected 9:3:3:1 ratio (Fig. S1). QTL mapping on the presence or absence of white head spotting in the F_2_ mice identified a significant QTL on chromosome 10 (Fig. S1). This interval contained the kit ligand (*Kitl*; stem cell factor), which was previously shown to underlie this trait in the pre-CC population (44).

10.1128/mBio.02791-19.1 FIG S1 Description of F_2_ cross population and test scan for the Mendelian blaze trait. (A) Coat color phenotypes of parent mice, CC001 (black), CC042 (agouti with white head blaze), F_1_ (agouti), and F_2_ (agouti, black, agouti and head blaze, black and head blaze) mice. (B) Genetic map of the F_2_ population used in QTL studies. Vertical lines show chromosomes (1 to 19 autosomes; X, X chromosome). Horizontal ticks show marker locations (in centimorgans). (C) Number of male (M) versus female (F) mice. (D) Number of mice with no head blaze (−) versus head blaze (+) in F_2_ mice. (E) Results of genome scan for blaze trait. (F) Bayes interval for the blaze trait on chromosome 10 (shaded), containing *Kitl*, previously shown to be associated with the WSB^blaze^ phenotype (44). Download FIG S1, PDF file, 1.0 MB. Copyright © 2019 Smith et al. 2019 Smith et al. This content is distributed under the terms of the Creative Commons Attribution 4.0 International license.

**FIG 4:**
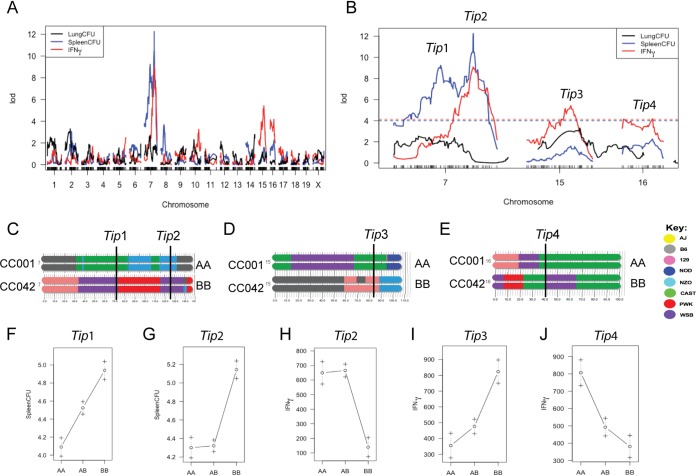
QTL mapping identifies four loci underlying TB susceptibility. (A, B) QTL scans of the numbers of lung CFU, the numbers of spleen CFU, and IFN-γ abundance in the lung identify four tuberculosis immunophenotype (*Tip*) loci on chromosomes 7, 15, and 16. The dashed lines in panel B represent a 5% false discovery rate for each trait based on permutation analysis. (C to E) The *Tip* loci on each chromosome are indicated at the marker with the peak LOD. (F to J) Allele effect plots for the indicated *Tip* loci. One hundred seventy F_2_ mice (86 female and 84 male mice) were successfully genotyped and used for QTL mapping.

Next, we conducted QTL mapping on the tuberculosis-associated phenotypes, consisting of the numbers of lung CFU, numbers of spleen CFU, and IFN-γ levels. Using batch and sex as covariates, we identified four significant QTL that affect the measured tuberculosis immunophenotypes (*Tip1* to *Tip4*) (Table 1 and Fig. 4A and B).

**TABLE 1:** Tuberculosis immunophenotype (*Tip*) QTL in a (CC001 × CC042)F_2_ cross^*a*^

QTL	Chromosome	Trait	*P* value	Max LOD	Peak marker	Peak (Mb)	Bayes interval (Mb)	Haplotype		Inheritance mode	Variance (%)
								Low	High		
*Tip1*	7	No. of spleen CFU	<10^−4^	9.2	gUNC13104259	72.1	3.6–72.9	CAST	WSB	Additive	17.6
*Tip2*	7	No. of spleen CFU	<10^−4^	12.3	gUNC13793270	125.4	124.7–127.3	NZO	WSB	Recessive	22.5
*Tip2*	7	IFN-γ level	7 × 10^−4^	9.1	gUNC13793270	125.4	121.0–130.7	NZO	WSB	Recessive	21.4
*Tip3*	15	IFN-γ level	0.011	5.4	mbackupUNC150396514	83.1	53.7–89.7	CAST	129	Additive (nonlinear)	13.5
*Tip4*	16	IFN-γ level	0.046	4.2	UNC26693650	40.8	4.5–44.7	WSB	CAST	Additive (nonlinear)	10.6

a Low and high haplotypes are provided at the peak logarithm of the odds (LOD) of each QTL. High and low haplotypes are provided relative to each specific trait. *P* values were determined by the permutation test. The fraction of the variance explained by each QTL was estimated by fitting a single QTL model for each trait at the respective peak locations with sex and batch as covariates. NZO, New Zealand Obese.

The numbers of spleen CFU mapped to two distinct QTL on chromosome 7: proximal *Tip1* at 72 Mb and distal *Tip2* at 125 Mb (Fig. 4C). *Tip1* was inherited in an additive fashion, with heterozygous mice carrying both the castaneous (CAST)/EiJ (CAST) and WSB haplotypes exhibiting a phenotype intermediate to the phenotypes of both homozygotes (Fig. 4F). In contrast, the susceptibility phenotype associated with *Tip2* was recessive, with mice homozygous for the WSB allele demonstrating a 10-fold increase in the number of spleen CFU, on average (Fig. 4G). IFN-γ production in the lung was associated with 3 distinct QTL. The main locus explained 21.4% of the variation and was mapped to the *Tip2* region on chromosome 7 (Fig. 4B), evidence that the same variant likely controls IFN-γ and the bacterial burden at this locus. Two additional QTL were also associated with IFN-γ levels, mapping to chromosomes 15 and 16 (*Tip3* and *Tip4*, respectively) (Fig. 4A and B). A second potential QTL on proximal chromosome 16 was also associated with IFN-γ; however, LOD did not reach genome-wide significance at a *P* value of <0.05. At *Tip3*, low IFN-γ levels were associated with haplotypes from the CAST founder (Fig. 4D), a strain previously found to lack IFN-γ expression in the lung upon infection with either M. tuberculosis or poxvirus (41, 45, 46). Notably, *Tip1* was associated with the numbers of CFU but not with IFN-γ levels, indicating that this variant was functionally distinct from *Tip2*. No QTL were associated with the numbers of lung CFU, suggesting that this trait is under more complex genetic control than the others. In sum, these functionally and genetically diverse QTL indicated that the immune response to M. tuberculosis was under multigenic control in these strains.

Considering that both *Tip1* and *Tip2* are on chromosome 7 and are driven by the WSB parent haplotype, we tested the independence of these peaks by remapping the number of spleen CFU using the genotypes at *Tip1* as a covariate. After removing the variation explained by the proximal *Tip1* QTL, the distal *Tip2* QTL still met the threshold for genome-wide significance (Fig. S2A). In addition, fitting of a three QTL model to the number of spleen CFU phenotypes showed that *Tip1*, *Tip2*, and *Tip3* all contributed additively; removing any of them from the full model resulted in a significantly poorer fit (Fig. S2B). Altogether, we identified four independent QTL in the F_2_ cross, indicating multigenic control of M. tuberculosis immunity in this F_2_ population.

10.1128/mBio.02791-19.2 FIG S2 Tests for independence of *Tip1* and *Tip2* QTL underlying the numbers of spleen CFU. (A) The number of spleen CFU trait was remapped using the genotype probabilities at *Tip1* and *Tip2* separately as covariates. The distal QTL (*Tip2*) reaches a significant LOD after the variation explained by *Tip1* was removed and vice versa. (B) A multi-QTL model including *Tip1*, *Tip2*, and *Tip3* was fit for the number of spleen CFU phenotype (batch and sex are included as covariates). The table shows the results from drop-one-term analysis of variance, where each QTL is dropped from the model, one at a time, and the submodel with that factor omitted is compared to the full model. The results provide substantial evidence for all QTL. Column headings: df, number of degrees of freedom; Type III SS, type III sum of squares; LOD, LOD score; %var, percentage of variance explained; Pvalue(Chi2), *P* value for chi square; Pvalue(F), *P* value for the *F* distribution. The values for the number of degrees of freedom, type III sum of squares, LOD score, and percentage of variance explained are those used to compare the full model to the submodel. Download FIG S2, PDF file, 0.1 MB. Copyright © 2019 Smith et al. 2019 Smith et al. This content is distributed under the terms of the Creative Commons Attribution 4.0 International license.

### T cell function and recruitment are impaired in CC042 mice.

**FIG 5:**
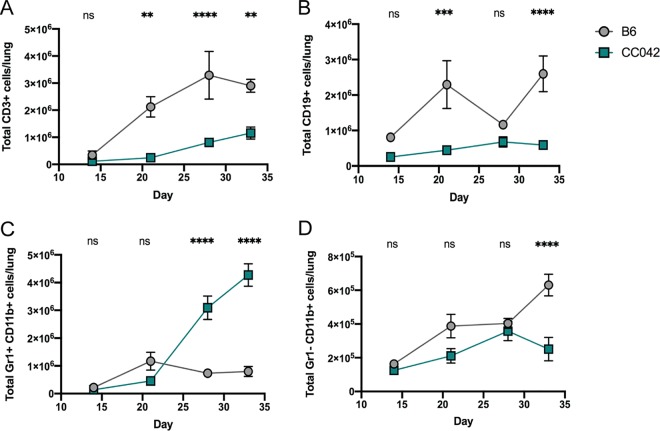
The susceptibility of CC042 mice correlates with altered numbers of lung leukocytes. The total numbers of the following cells were enumerated in the lungs of B6 and CC042 mice: T cells (lymphocytes > single cells > CD3^+^ CD19^−^ cells) (A), B cells (lymphocytes > single cells > CD3^−^ CD19^+^ cells) (B), neutrophils (single cells > Gr1^+^ CD11b^+^ cells) (C), and monocytes/macrophages (single cells > Gr1^−^ CD11b^+^ cells) (D). All mice were infected in one batch, and 3 males and 3 females of each strain were used for analysis at each time point. The data in the graphs represent the mean ± SD. One-way analysis of variance with Sidak’s multiple-comparison test was used to determine significance. **, *P* < 0.01; ***, *P* < 0.001; ****, *P* < 0.0001; ns, not significant.

Concurrently with our genetic mapping strategy, we enumerated changes in leukocyte cell types that could alter the susceptibility of CC042 mice relative to B6 mice. The accumulation of T and B lymphocytes in the lungs of B6 mice began between 14 and 21 days postinfection (Fig. 5A and B). In contrast, the numbers of T and B cells in the lungs of CC042 mice were significantly reduced. This paucity of lymphocyte accumulation in the lungs of CC042 mice was mirrored by a dramatic increase in CD11b^+^ Gr1^+^ granulocytes (Fig. 5C), consistent with the neutrophilic infiltrates observed in the lung histopathology (Fig. 2). Significant differences in the CD11b^+^ Gr1^−^ monocyte/macrophage subset between these mice was apparent only at the last time point, at which time the CC042 animals had become moribund (Fig. 5D).

**FIG 6:**
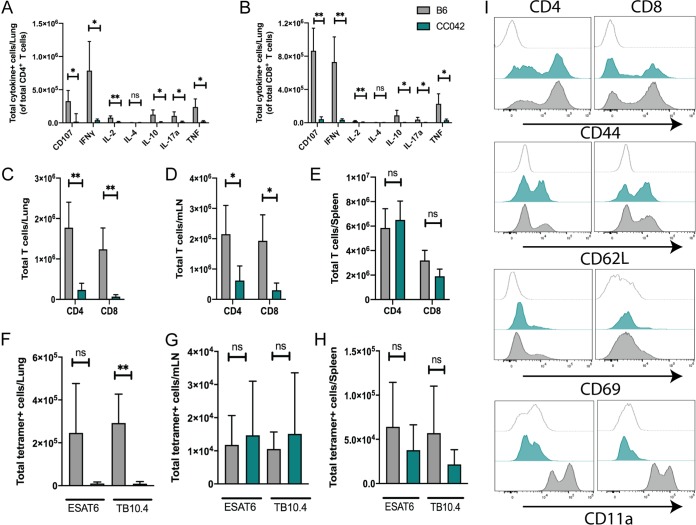
CC042 mice have a defect in T cell recruitment to the lung and lack CD11a expression. (A, B) Intracellular cytokine staining (ICS) of CD4 (A) and CD8 (B) T cells reveals a defect in the number of cytokine-producing T cells in the lungs of CC042 mice. (C to E) Total number of CD4 and CD8 T cells in the lung (C), mediastinal lymph node (D), and spleen (E). (F to H) Total number of ESAT-6 (CD4) or TB10.4 (CD8) tetramer-positive cells in the lung (F), mediastinal lymph node (G), and spleen (H). Bar plots show the mean + SD. Welch’s *t* test was used to determine significance. *, *P* < 0.05; **, *P* < 0.01; ns, not significant. (I) Histograms of CD4 (left) and CD8 (right) T cells stained for activation and migration markers CD44, CD62L, CD69, and CD11a for the isotype control (top, light gray trace), CC042 mice (middle, teal trace), and B6 mice (bottom, gray trace).

The reduction in IFN-γ production and the paucity of pulmonary T cells in the lungs of CC042 mice suggested that their susceptibility to M. tuberculosis might be related to a defect in T cell function. To assess effector function, we isolated cells from the lungs of infected B6 and CC042 mice and stimulated them with anti-CD3, to determine whether T cells from CC042 mice had differentiated into a distinct T cell subset (e.g., Th1 versus Th2, Th17, and regulatory T cells). Using a standard intracellular cytokine staining (ICS) approach, we found fewer CD4 and CD8 T cells from CC042 mice that produced IFN-γ, tumor necrosis factor (TNF), interleukin-17a (IL-17a), IL-2, IL-10, and CD107a upon stimulation than T cells from B6 mice (Fig. 6A and B). Thus, instead of representing an altered T cell differentiation state, the lack of cytokine production by T cells from the lungs of infected CC042 mice indicated that there is an impairment in either T cell priming or recruitment of T cells to the lung.

In order to distinguish between these possibilities, we counted the number of CD4 and CD8 T cells in the lung, mediastinal lymph node (mLN), and spleen at 28 days postinfection using flow cytometry. At the same time, we enumerated the number of antigen-specific CD4 and CD8 T cells using ESAT-6 (CD4) and TB10.4 (CD8) tetramers. CC042 mice had fewer total CD4 and CD8 T cells in the lung than B6 mice (Fig. 6C). This was also true in the mLN but not the spleen (Fig. 6D and E). While we saw that CC042 mice had significantly fewer ESAT-6-specific CD4 T cells and TB10.4-specific CD8 T cells in the lung than B6 animals, both groups of mice had similar numbers of antigen-specific T cells in the mLN and the spleen (Fig. 6F to H). When antigen-specific T cells were considered as a fraction of the total number of T cells, the frequencies of ESAT-6-specific T cells was similar in the lung, spleen, and mLN of B6 and CC042 mice, while the frequency of TB10.4-specific T cells was lower in the lungs of CC042 mice than in those of B6 mice but higher in the spleen and mLN of CC042 mice than in those of B6 mice (Fig. S3). Taken together, this suggests that T cell priming of M. tuberculosis-specific CD4 and CD8 T cells was occurring in the draining lymph node and that the diminished T cell numbers in the lungs of CC042 mice is due to an impairment in T cell recruitment. As such, we examined the cell surface expression of CD44, CD69, CD62L, and CD11a on CD4 and CD8 T cells from the lungs of infected CC042 and B6 mice, as these markers have been classically associated with either T cell activation or migration (47, 48). We found that CD4 and CD8 T cells from the lungs of CC042 and B6 mice had appropriately upregulated CD44 and CD69 and downregulated CD62L (Fig. 6I). However, CD11a was undetectable on both CD4 and CD8 T cells from CC042 mice. As CD11a is the αL component of αLβ2, the principal β2-integrin on T cells that is crucial for lymphocyte trafficking, this defect could explain many of the immunological differences observed between CC042 and B6 mice.

10.1128/mBio.02791-19.3 FIG S3 Frequency of antigen-specific T cells in B6 and CC042 mice following pulmonary M. tuberculosis infection. Representative flow plots showing the frequencies of ESAT-6-specific CD4 T cells and TB10.4-specific CD8 T cells in the lung (A), mediastinal lymph node (mLN) (B), and spleen (C) of B6 (gray shading) and CC042 (teal shading) mice at 4 weeks after pulmonary M. tuberculosis infection. Bar plots show the mean ± SD for ESAT-6-specific CD4 T cells and TB10.4-specific CD8 T cells in the lung (D), mLN (E), and spleen (F) at the same time point. Sidak’s multiple-comparison test was used to determine significance. *, *P* < 0.05; ***, *P* < 0.001. Download FIG S3, PDF file, 0.5 MB. Copyright © 2019 Smith et al. 2019 Smith et al. This content is distributed under the terms of the Creative Commons Attribution 4.0 International license.

### A CC042 private mutation in *Itgal* explains *Tip2*-driven susceptibility.

**FIG 7:**
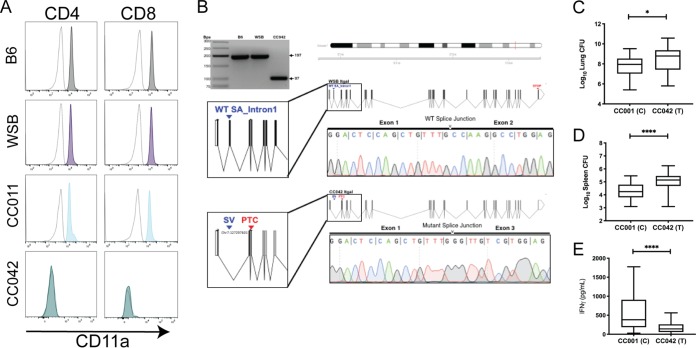
A private mutation in *Itgal* in CC042 mice explains the *Tip2*-driven susceptibility. (A) Histograms depicting CD11a staining in CD4 (left) and CD8 (right) T cells from B6 (gray shading), WSB (purple shading), CC011 (light blue shading), and CC042 (teal shading) mice. The isotype control antibody staining is shown on each plot as a dotted gray trace. (B) PCR primers flanking the putative private *Itgal* mutation were used to amplify cDNA from B6, WSB, and CC042 mouse RNA. PCR products were separated by gel electrophoresis on a 3% agarose gel. The second and third lanes show for B6 band WSB (the parental allele for CC042), respectively, the 197-bp amplicons. The 100-bp size decrease in the CC042 mouse-derived product is consistent with the loss of exon 2 (fourth lane and schematic). Sanger sequencing traces from the CC042 and WSB mouse amplicons are shown. WT, wild type; SA, splice acceptor; SV, splice variant; PTC, premature termination codon. (C to E) TB immunophenotypes were reevaluated in F_2_ progeny of the CC001 × CC042 mice that were homozygous for each parental allele at the *Itgal* locus (probe UNC13811649): numbers of lung CFU (C), numbers of spleen CFU (D), and lung IFN-γ levels (E). Welch’s *t* test was used to determine significance. *, *P* < 0.05; ****, *P* < 0.0001. Box-and-whiskers plots indicate the median and minimum-maximum values.

The gene encoding CD11a, *Itgal*, is located on chromosome 7, within the *Tip2* locus identified in our intercross. The lack of CD11a expression on CC042 lymphocytes implicated *Itgal* variation as the basis for *Tip2*. To investigate this possibility, CD11a expression was assayed in the WSB and CC011 strains, which contain the susceptibility-associated WSB haplotype at *Tip2* (Fig. 7A). We found that WSB and CC011 mouse splenocytes expressed CD11a levels similar to those expressed by B6 mouse splenocytes, leading us to hypothesize that CC042 mice had incurred a private mutation during inbreeding that impacts CD11a production.

Using the whole-genome sequences of representative CC strains, variants private to each have been identified (43, 49). Using this data set, we found that the CC042 mouse genome sequence contains a 15-bp deletion in the first intron of the *Itgal1* gene which is not present in the ancestral WSB mouse allele. This deletion alters the canonical splice acceptor sequence (AG) at the 3′ terminus of intron 1 (http://Jul2019.archive.ensembl.org/Mus_musculus_WSB_EiJ/Transcript/Exons?db=core;g=MGP_WSBEiJ_G0032170;r=7:131372531-131430694;t=MGP_WSBEiJ_T0085398). Although the resultant mutant sequence (TG) has been observed to function as a splice acceptor in certain transcripts (50), we hypothesized that this mutation could alter splicing. Amplification and sequencing of a fragment spanning exons 1 and 3 of the *Itgal* mRNA confirmed that the CC042 mouse transcript lacked the exon 2 sequence that was contained in both the B6 and WSB mouse mRNAs (Fig. 7B). The lack of exon 2 is predicted to produce a frameshift and premature termination (Fig. 7B).

While *Tip2* was significantly associated only with the number of spleen CFU and lung IFN-γ levels in the whole-genome scans, the lack of T cells in the lungs of CC042 animals suggested that the CD11a deficiency also influenced bacterial replication at this site. To test this hypothesis, we used the measured phenotypes from the F_2_ cross and found that the CC042 *Itgal* allele was significantly associated with higher numbers of CFU in both lung and spleen, as well as IFNγ production (Fig. 7C to E). This analysis indicated that the *Itgal* mutation affects all metrics of tuberculosis susceptibility in CC042 mice.

## DISCUSSION

In this work, we used a classic genetic strategy to investigate the diversity of responses to M. tuberculosis infection observed in CC strains. Previous work identified a number of genetically dissociable immunophenotypes in M. tuberculosis-infected CC founder lines, including bacterial load and IFN-γ production (41). The intercross between two phenotypically divergent CC strains reported here supports the independence of these traits and demonstrates that multiple phenotypes can be mapped simultaneously using this strategy. Investigating the genetic architecture of TB disease in these highly diverse mice revealed a number of new insights that would not have been apparent in intercrosses between more genetically homogeneous lab strains.

A loss-of-function mutation in *Itgal* was found to account for the *Tip2* QTL and explain a significant portion of the susceptibility of the CC042 line. This finding is consistent with that of a concurrent study that also identified *Itgal* to be underlying the susceptibility of CC042 mice to Salmonella enterica serovar Typhimurium (51). We previously found that *Itgal* deletion in the B6 mouse background resulted in a defect in T cell recruitment to the lung and an increased pulmonary M. tuberculosis burden (21); both of these traits are associated with *Itgal* deficiency in the CC001 × CC042 intercross. While *Tip2* was significantly associated only with the number of spleen CFU and IFN-γ production in whole-genome scans, the *Itgal* genotype also correlated with the number of lung CFU when examined in isolation. Thus, we conclude that *Itgal* affects pathogenesis in both lung and spleen and that the lack of a genome-wide association between *Itgal* and the number of lung CFU is likely due to complex genetic factors, which could include interactive effects with sex and the presence of additional variants that dilute the effect of the *Itgal* mutation. In addition, the observation that the number of lung CFU in F_1_ mice exceeds the range found in either parent strain suggests a particularly complex mode of inheritance that could involve both genetic and nongenetic factors, such as parental imprinting. Further studies, including reciprocal crosses to account for epigenetic effects, will be necessary to understand the genetic basis of this trait.

While the disease-promoting effect of CD11a deficiency was generally consistent between the B6 background and the recombinant CC genotype, the loss of *Itgal* is insufficient to explain the susceptibility of the CC042 strain. These CC042 mice succumb to infection approximately 5 months earlier than *Itgal^−/−^* B6 mice (21), likely due to the more dramatic infiltration of granulocytes and the necrosis observed in the lung. The increased susceptibility of CC042 animals can be attributed, at least in part, to *Tip1*. The variant(s) underlying *Tip1* appears to be functionally distinct from *Itgal*, as the effects of *Tip1* and *Tip2* were additive, these traits differed in their mode of inheritance, and *Tip1* was not associated with IFN-γ levels. These data indicate that IFN-γ-independent mechanisms, such as those underlying *Tip1*, can act in an additive fashion to increase the susceptibility of animals with a more canonical immunodeficiency that affects Th1 cell activity.

This intercross between two very diverse genotypes allowed the mapping of QTL that may be associated with an additional trait previously identified in the CAST founder strain. Despite being relatively resistant to M. tuberculosis infection, CAST mice do not produce detectable levels of IFN-γ in the lung (41). This observation is one of many indicating that IFN-γ-independent immune mechanisms play an important protective role, particularly in the lungs (27, 28, 52–54). *Tip1* and *Tip3* are likely related to the IFN-γ-deficient phenotype of CAST mice. This founder haplotype at *Tip3* is associated with low levels of IFN-γ production, and the CAST allele at *Tip1* reduces the number of CFU without influencing IFN-γ levels. Defining the basis of this phenotype might represent an important step in understanding the immune response to M. tuberculosis in the recently identified subset of humans that control M. tuberculosis infection in the absence of a detectable IFN-γ response (27).

The CC population was initially envisioned as a genetic mapping resource, based on the random distribution of founder alleles between strains (55). While the CC panel has been shown to be valuable for this type of study, the *Itgal* mutation identified in this work was not derived from a founder line and likely occurred during the process of inbreeding CC042 mice. Such mutations in individual CC strains provide an additional advantage to studies within the CC: both common variants and private variants (∼28,000) circulate in this population and can drive disease responses (43, 49). While these mutations are invisible in genetic association studies that are based on a comparison of CC lines, our work shows that their effect can be revealed through intercrosses and that the molecular characterization of these relatively rare variants can be rapidly achieved. This feature, in combination with the variety of phenotypes that can be addressed in CC × CC intercrosses, highlights the value of this approach.

The immune response to M. tuberculosis in natural populations is variable. Several lines of recent evidence suggest the importance of mechanisms distinct from canonical Th1 immunity that dominate in the classic mouse model of TB, which relies on a small number of genetically similar mouse lines. Using a simple intercross strategy, we leveraged the genetic diversity of CC lines to define chromosomal loci controlling three distinct TB-related traits: *Itgal*-dependent T cell recruitment (*Tip2*), IFN-γ-independent bacterial control (*Tip1*), and IFN-γ production (*Tip3* and *Tip4*). The dissociation of bacterial control from IFN-γ production was likely facilitated by the presence of haplotypes that are absent from standard mouse strains, supporting the value of genetic diversity to understand highly variable traits, such as TB susceptibility.

## MATERIALS AND METHODS

### Ethics statements and experimental animals.

C57BL/6J (Jax stock number 0664) (B6) mice were purchased from The Jackson Laboratory. CC042/GeniUnc and CC001/Unc mice were obtained from the Systems Genetics Core Facility at the University of North Carolina (56) and bred at the University of Massachusetts (UMass) Medical School under specific-pathogen-free conditions and in accordance with the University of Massachusetts Medical School IACUC guidelines. F_1_ mice were generated from crossing CC001 females with CC042 males (CC001 × CC042). The F_2_ mice used for QTL mapping were obtained from crossing these F_1_ mice; e.g., all F_2_ animals were [(CC001 × CC042) × (CC001 × CC042)]F_2_. Two hundred one F_2_ mice were made and phenotyped, and 170 were successfully genotyped and used for QTL analysis (86 female and 84 male mice). Both female and male mice were used throughout the study, as indicated throughout the text. All animals used for experiments were 8 to 12 weeks old.

### Mycobacterium tuberculosis infection.

Wild-type M. tuberculosis strain H37Rv (phthiocerol dimycocerosate positive) was used for all studies. Prior to infection, the bacteria were cultured in 7H9 medium containing 10% oleic acid-albumin-dextrose-catalase (OADC) growth supplement enrichment (Becton, Dickinson) and 0.05% Tween 80. For aerosol infections, bacteria were resuspended in phosphate-buffered saline (PBS) containing Tween 80 (PBS-T). Prior to infection, the bacteria were sonicated and then delivered via the respiratory route using an aerosol generation device (Glas-Col). Groups of mice were sacrificed at 24 h postinfection to enumerate the infectious dose. The infectious dose for all experiments ranged from 50 to 150 CFU.

### CFU enumeration and cytokine quantification.

To determine the number of CFU, mice were anesthetized via inhalation with isoflurane (Piramal) and euthanized via cervical dislocation. The organs were aseptically removed and individually homogenized, and viable bacteria were enumerated by plating 10-fold serial dilutions onto 7H10 agar plates. The plates were incubated at 37°C, and the colonies were counted after 21 days. Cytokine concentrations in cell-free lung homogenates were quantified using commercial enzyme-linked immunosorbent assay (ELISA) kits (IFN-γ Duo Set; catalog number DY485; R&D Systems) according to the manufacturer’s instructions.

### Histology.

Lung lobes from B6 and CC042 mice infected with M. tuberculosis were fixed in 10% neutral buffered formalin, embedded in paraffin, and sectioned at 5 μm. The sections were stained with hematoxylin and eosin (H&E). All sectioning and staining were done by the Diabetes and Endocrinology Research Center Morphology Core (DERC) at the University of Massachusetts Medical School. Images were captured on a TissueGnostics TissueFAXS Plus slide scanning microscope at ×2 and ×20 magnifications.

### Flow cytometry analysis.

Lung tissue was harvested in RPMI containing fetal bovine serum (FBS) and placed in C tubes (Miltenyi). Collagenase type IV/DNase I was added, and the tissues were dissociated for 10 s on a GentleMACS system (Miltenyi). The tissues were incubated for 30 min at 37°C with oscillations and then dissociated for an additional 30 s on a GentleMACS system. Lung homogenates were passed through a 70-μm-pore-size filter. Cell suspensions were washed in RPMI, passed through a 40-μm-pore-size filter, and aliquoted into 96-well plates for flow cytometry staining. Nonspecific antibody binding was first blocked using the Fc Block reagent, after which the cells were then stained with CD3-BV785 (clone 145-2CL1), CD8-allophycocyanin (APC)-Fire 750 (clone 53-8.7), CD44-peridinin chlorophyll protein-Cy5.5 (clone IM7), CD11a-BV711 (clone M17/4), CD69-phycoerythrin (PE)-Cy7 (clone H1.2F3), and CD62L-BV570 (clone MEL-14) from BioLegend and CD4-Alexa Fluor 700 (clone RM4-5) from BD Biosciences. In some experiments, intracellular cytokine staining was also performed. After surface marker staining, cells were subsequently permeabilized using Cytofix/Cytoperm solution (BD Biosciences) and stained with IFN-APC (clone XMG1.2), IL-2 PE-Cy7 (clone JES6-5H4), IL-17a-BV650 (clone TC11-18H10.1), TNF-BV421 (clone MP6-XT22), IL-10 (clone JES5-16E3), and CD107a-PE (clone 1D4B) from BioLegend and IL-4-Alexa Fluor 488 (clone 11B11) from Invitrogen. Live cells were identified using fixable Live/Dead Aqua stain (Life Technologies). The cells were stained for 30 min at room temperature and fixed in 1% paraformaldehyde for 60 min. All flow cytometry assays were run on either a MACSQuant Analyzer 10 (Miltenyi) or Aurora (Cytek) flow cytometer, and the results were analyzed using FlowJo (version 10) software (TreeStar).

### Genotyping and QTL mapping.

DNA (1,500 ng) was genotyped by Neogen Inc. using the MiniMUGA array. We filtered markers to those that were consistent within a previously published set of CC042 and CC001 mouse genotypes (43) and diagnostic between these strains (i.e., a CC001 genotype that was not equal to the CC042 genotype and F_1_ genotype was called heterozygous). After finding and removing misplaced markers, identified using the droponemarker function of the R package qtl (R/qtl), regions of dense marker coverage were thinned to a spacing of 0.1 cM. The final genetic map contained 1,806 markers. Genotype and phenotype data are available in Tables S1 and S2 in the supplemental material.

10.1128/mBio.02791-19.4 TABLE S1 Phenotype data for F_2_ intercross mice. Download Table S1, XLSX file, 0.07 MB. Copyright © 2019 Smith et al. 2019 Smith et al. This content is distributed under the terms of the Creative Commons Attribution 4.0 International license.

10.1128/mBio.02791-19.5 TABLE S2 MiniMUGA genotype data for CC001, CC042, and F_2_ intercross mice. Download Table S2, XLSX file, 1.8 MB. Copyright © 2019 Smith et al. 2019 Smith et al. This content is distributed under the terms of the Creative Commons Attribution 4.0 International license.

Genotype and phenotype data were imported into R (version 3.4.3) and reformatted for R/qtl (version 1.42-8). Genotype probabilities were calculated at a 0.25-cM spacing, and QTL mapping was carried out using the scanone function and batch and sex as additive covariates. Significant LOD thresholds were established by a permutation test with 10,000 permutation replicates. Multi-QTL models were fit using R/qtl’s fitqtl function. LOD profiles and effect plots were generated using the plotting functions of the R/qtl package.

### *Ex vivo* RNA isolation and qRT-PCR.

Bone marrow-derived macrophages (BMDMs) from B6, WSB, and CC042 mice were generated. Briefly, marrow was isolated from the femurs and tibias of age- and sex-matched mice and cultured in high-glucose Dulbecco modified Eagle medium (catalog number 11965092; Gibco) supplemented with l-glutamine, 10% fetal bovine serum (FBS; catalog number F4135; Sigma), and 20% L929 conditioned medium. After 7 days, differentiated cells were lifted with PBS with 10 mM EDTA and seeded for subsequent experimentation. RNA was isolated by lysing the cells in the TRIzol reagent (catalog number 15596018; Thermo Fisher) and purified using a Direct-zol RNA Miniprep plus kit (catalog number R2070; Zymo Research) per the manufacturer’s recommendations. Following RNA quantification with a NanoDrop spectrophotometer, samples were diluted to 5 ng/μl and used for quantitative reverse transcription-PCR (qRT-PCR) with a Luna One-Step Universal quantitative PCR kit (catalog number E3005; New England Biolabs). Gene-specific primers for target transcripts were used at a final concentration of 400 μM with 15 ng of RNA. Primer sequences for Itgal (primer RT-Itgal_1F, 5′-CCAGACTTTTGCTACTGGGAC-3′; primer RT-Itgal_1R, 5′-GCTTGTTCGGCAGTGATAGAG-3′) were designed using publicly available genomic sequences for B6, WSB, and CC042 mice (49). Itgal PCR products were separated by gel electrophoresis on a 3% agarose gel, and their sequences were determined by Sanger sequencing.

### Statistical analysis.

Statistical tests were performed using GraphPad Prism (version 7) software. The correlation between measured traits was visualized using the chart correlation function in the PerformanceAnalytics package in R (version 3.2.4) software.

### Data availability.

All relevant data to support the findings of this study are located within the paper and supplemental files.

## References

[1] Houben R, Dodd PJ 2016 The global burden of latent tuberculosis infection: a re-estimation using mathematical modelling. PLoS Med 13:e1002152. doi:10.1371/journal.pmed.1002152. 27780211 PMC5079585

[2] Comstock GW 1978 Tuberculosis in twins: a re-analysis of the Prophit survey. Am Rev Respir Dis 117:621–624. doi:10.1164/arrd.1978.117.4.621. 565607

[3] Kallmann FJ 1943 Genetic mechanisms in resistance to tuberculosis. Psych Q 17:32–37. doi:10.1007/BF01744162.

[4] Bogunovic D, Byun M, Durfee LA, Abhyankar A, Sanal O, Mansouri D, Salem S, Radovanovic I, Grant AV, Adimi P, Mansouri N, Okada S, Bryant VL, Kong X-F, Kreins A, Velez MM, Boisson B, Khalilzadeh S, Ozcelik U, Darazam IA, Schoggins JW, Rice CM, Al-Muhsen S, Behr M, Vogt G, Puel A, Bustamante J, Gros P, Huibregtse JM, Abel L, Boisson-Dupuis S, Casanova J-L 2012 Mycobacterial disease and impaired IFN-γ immunity in humans with inherited ISG15 deficiency. Science 337:1684–1688. doi:10.1126/science.1224026. 22859821 PMC3507439

[5] Bustamante J, Arias AA, Vogt G, Picard C, Galicia LB, Prando C, Grant AV, Marchal CC, Hubeau M, Chapgier A, de Beaucoudrey L, Puel A, Feinberg J, Valinetz E, Jannière L, Besse C, Boland A, Brisseau J-M, Blanche S, Lortholary O, Fieschi C, Emile J-F, Boisson-Dupuis S, Al-Muhsen S, Woda B, Newburger PE, Condino-Neto A, Dinauer MC, Abel L, Casanova J-L 2011 Germline CYBB mutations that selectively affect macrophages in kindreds with X-linked predisposition to tuberculous mycobacterial disease. Nat Immunol 12:213–221. doi:10.1038/ni.1992. 21278736 PMC3097900

[6] Filipe-Santos O, Bustamante J, Chapgier A, Vogt G, de Beaucoudrey L, Feinberg J, Jouanguy E, Boisson-Dupuis S, Fieschi C, Picard C, Casanova J-L 2006 Inborn errors of IL-12/23- and IFN-gamma-mediated immunity: molecular, cellular, and clinical features. Semin Immunol 18:347–361. doi:10.1016/j.smim.2006.07.010. 16997570

[7] Hambleton S, Salem S, Bustamante J, Bigley V, Boisson-Dupuis S, Azevedo J, Fortin A, Haniffa M, Ceron-Gutierrez L, Bacon CM, Menon G, Trouillet C, McDonald D, Carey P, Ginhoux F, Alsina L, Zumwalt TJ, Kong X-F, Kumararatne D, Butler K, Hubeau M, Feinberg J, Al-Muhsen S, Cant A, Abel L, Chaussabel D, Doffinger R, Talesnik E, Grumach A, Duarte A, Abarca K, Moraes-Vasconcelos D, Burk D, Berghuis A, Geissmann F, Collin M, Casanova J-L, Gros P 2011 IRF8 mutations and human dendritic-cell immunodeficiency. N Engl J Med 365:127–138. doi:10.1056/NEJMoa1100066. 21524210 PMC3136554

[8] Salem S, Gros P 2013 Genetic determinants of susceptibility to mycobacterial infections: IRF8, a new kid on the block. Adv Exp Med Biol 783:45–80. doi:10.1007/978-1-4614-6111-1_3. 23468103

[9] Bellamy R, Ruwende C, Corrah T, McAdam KP, Whittle HC, Hill AV 1998 Variations in the NRAMP1 gene and susceptibility to tuberculosis in West Africans. N Engl J Med 338:640–644. doi:10.1056/NEJM199803053381002. 9486992

[10] Curtis J, Luo Y, Zenner HL, Cuchet-Lourenço D, Wu C, Lo K, Maes M, Alisaac A, Stebbings E, Liu JZ, Kopanitsa L, Ignatyeva O, Balabanova Y, Nikolayevskyy V, Baessmann I, Thye T, Meyer CG, Nürnberg P, Horstmann RD, Drobniewski F, Plagnol V, Barrett JC, Nejentsev S 2015 Susceptibility to tuberculosis is associated with variants in the ASAP1 gene encoding a regulator of dendritic cell migration. Nat Genet 47:523–527. doi:10.1038/ng.3248. 25774636 PMC4414475

[11] Thye T, Owusu-Dabo E, Vannberg FO, van Crevel R, Curtis J, Sahiratmadja E, Balabanova Y, Ehmen C, Muntau B, Ruge G, Sievertsen J, Gyapong J, Nikolayevskyy V, Hill PC, Sirugo G, Drobniewski F, van de Vosse E, Newport M, Alisjahbana B, Nejentsev S, Ottenhoff THM, Hill AVS, Horstmann RD, Meyer CG 2012 Common variants at 11p13 are associated with susceptibility to tuberculosis. Nat Genet 44:257–259. doi:10.1038/ng.1080. 22306650 PMC3427019

[12] Thye T, Vannberg FO, Wong SH, Owusu-Dabo E, Osei I, Gyapong J, Sirugo G, Sisay-Joof F, Enimil A, Chinbuah MA, Floyd S, Warndorff DK, Sichali L, Malema S, Crampin AC, Ngwira B, Teo YY, Small K, Rockett K, Kwiatkowski D, Fine PE, Hill PC, Newport M, Lienhardt C, Adegbola RA, Corrah T, Ziegler A, Morris AP, Meyer CG, Horstmann RD, Hill A 2010 Genome-wide association analyses identifies a susceptibility locus for tuberculosis on chromosome 18q11.2. Nat Genet 42:739–741. doi:10.1038/ng.639. 20694014 PMC4975513

[13] Medina E, North RJ 1998 Resistance ranking of some common inbred mouse strains to Mycobacterium tuberculosis and relationship to major histocompatibility complex haplotype and Nramp1 genotype. Immunology 93:270–274. doi:10.1046/j.1365-2567.1998.00419.x. 9616378 PMC1364188

[14] Cooper AM, Dalton DK, Stewart TA, Griffin JP, Russell DG, Orme IM 1993 Disseminated tuberculosis in interferon gamma gene-disrupted mice. J Exp Med 178:2243–2247. doi:10.1084/jem.178.6.2243. 8245795 PMC2191280

[15] Flynn JL, Chan J, Triebold KJ, Dalton DK, Stewart TA, Bloom BR 1993 An essential role for interferon gamma in resistance to Mycobacterium tuberculosis infection. J Exp Med 178:2249–2254. doi:10.1084/jem.178.6.2249. 7504064 PMC2191274

[16] MacMicking JD, Taylor GA, McKinney JD 2003 Immune control of tuberculosis by IFN-gamma-inducible LRG-47. Science 302:654–659. doi:10.1126/science.1088063. 14576437

[17] Schaible UE, Sturgill-Koszycki S, Schlesinger PH, Russell DG 1998 Cytokine activation leads to acidification and increases maturation of Mycobacterium avium-containing phagosomes in murine macrophages. J Immunol 160:1290–1296. 9570546

[18] Desvignes L, Ernst JD 2009 Interferon-gamma-responsive nonhematopoietic cells regulate the immune response to Mycobacterium tuberculosis. Immunity 31:974–985. doi:10.1016/j.immuni.2009.10.007. 20064452 PMC2807991

[19] Nandi B, Behar SM 2011 Regulation of neutrophils by interferon-γ limits lung inflammation during tuberculosis infection. J Exp Med 208:2251–2262. doi:10.1084/jem.20110919. 21967766 PMC3201199

[20] Mishra BB, Lovewell RR, Olive AJ, Zhang G, Wang W, Eugenin E, Smith CM, Phuah JY, Long JE, Dubuke ML, Palace SG, Goguen JD, Baker RE, Nambi S, Mishra R, Booty MG, Baer CE, Shaffer SA, Dartois V, McCormick BA, Chen X, Sassetti CM 2017 Nitric oxide prevents a pathogen-permissive granulocytic inflammation during tuberculosis. Nat Microbiol 2:17072. doi:10.1038/nmicrobiol.2017.72. 28504669 PMC5461879

[21] Ghosh S, Chackerian AA, Parker CM, Ballantyne CM, Behar SM 2006 The LFA-1 adhesion molecule is required for protective immunity during pulmonary Mycobacterium tuberculosis infection. J Immunol 176:4914–4922. doi:10.4049/jimmunol.176.8.4914. 16585587

[22] Berlin-Rufenach C, Otto F, Mathies M, Westermann J, Owen MJ, Hamann A, Hogg N 1999 Lymphocyte migration in lymphocyte function-associated antigen (LFA)-1-deficient mice. J Exp Med 189:1467–1478. doi:10.1084/jem.189.9.1467. 10224287 PMC2193056

[23] Slight SR, Khader SA 2013 Chemokines shape the immune responses to tuberculosis. Cytokine Growth Factor Rev 24:105–113. doi:10.1016/j.cytogfr.2012.10.002. 23168132 PMC3582802

[24] Walling BL, Kim M 2018 LFA-1 in T cell migration and differentiation. Front Immunol 9:952. doi:10.3389/fimmu.2018.00952. 29774029 PMC5943560

[25] Smith CM, Sassetti CM 2018 Modeling diversity: do homogeneous laboratory strains limit discovery? Trends Microbiol 26:892–895. doi:10.1016/j.tim.2018.08.002. 30166218 PMC6610874

[26] Roy Chowdhury R, Vallania F, Yang Q, Lopez Angel CJ, Darboe F, Penn-Nicholson A, Rozot V, Nemes E, Malherbe ST, Ronacher K, Walzl G, Hanekom W, Davis MM, Winter J, Chen X, Scriba TJ, Khatri P, Chien Y-H 2018 A multi-cohort study of the immune factors associated with M. tuberculosis infection outcomes. Nature 560:644–648. doi:10.1038/s41586-018-0439-x. 30135583 PMC6414221

[27] Lu LL, Smith MT, Yu KKQ, Luedemann C, Suscovich TJ, Grace PS, Cain A, Yu WH, McKitrick TR, Lauffenburger D, Cummings RD, Mayanja-Kizza H, Hawn TR, Boom WH, Stein CM, Fortune SM, Seshadri C, Alter G 2019 IFN-γ-independent immune markers of Mycobacterium tuberculosis exposure. Nat Med 25:977–987. doi:10.1038/s41591-019-0441-3. 31110348 PMC6559862

[28] Lu LL, Chung AW, Rosebrock TR, Ghebremichael M, Yu WH, Grace PS, Schoen MK, Tafesse F, Martin C, Leung V, Mahan AE, Sips M, Kumar MP, Tedesco J, Robinson H, Tkachenko E, Draghi M, Freedberg KJ, Streeck H, Suscovich TJ, Lauffenburger DA, Restrepo BI, Day C, Fortune SM, Alter G 2016 A functional role for antibodies in tuberculosis. Cell 167:433–443.e14. doi:10.1016/j.cell.2016.08.072. 27667685 PMC5526202

[29] Li H, Wang X-X, Wang B, Fu L, Liu G, Lu Y, Cao M, Huang H, Javid B 2017 Latently and uninfected healthcare workers exposed to TB make protective antibodies against Mycobacterium tuberculosis. Proc Natl Acad Sci U S A 114:5023–5028. doi:10.1073/pnas.1611776114. 28438994 PMC5441709

[30] Chackerian AA, Behar SM 2003 Susceptibility to Mycobacterium tuberculosis: lessons from inbred strains of mice. Tuberculosis (Edinb) 83:279–285. doi:10.1016/S1472-9792(03)00017-9. 12972341

[31] Logunova N, Korotetskaya M, Polshakov V, Apt A 2015 The QTL within the H2 complex involved in the control of tuberculosis infection in mice is the classical class II H2-Ab1 gene. PLoS Genet 11:e1005672. doi:10.1371/journal.pgen.1005672. 26618355 PMC4664271

[32] Kramnik I, Dietrich WF, Demant P, Bloom BR 2000 Genetic control of resistance to experimental infection with virulent Mycobacterium tuberculosis. Proc Natl Acad Sci U S A 97:8560–8565. doi:10.1073/pnas.150227197. 10890913 PMC26987

[33] Pan H, Yan B-S, Rojas M, Shebzukhov YV, Zhou H, Kobzik L, Higgins DE, Daly MJ, Bloom BR, Kramnik I 2005 Ipr1 gene mediates innate immunity to tuberculosis. Nature 434:767–772. doi:10.1038/nature03419. 15815631 PMC1388092

[34] Sissons J, Yan B-S, Pichugin AV, Kirby A, Daly MJ, Kramnik I 2009 Multigenic control of tuberculosis resistance: analysis of a QTL on mouse chromosome 7 and its synergism with sst1. Genes Immun 10:37–46. doi:10.1038/gene.2008.68. 18784733 PMC3060060

[35] Mitsos L-M, Cardon LR, Ryan L, LaCourse R, North RJ, Gros P 2003 Susceptibility to tuberculosis: a locus on mouse chromosome 19 (Trl-4) regulates Mycobacterium tuberculosis replication in the lungs. Proc Natl Acad Sci U S A 100:6610–6615. doi:10.1073/pnas.1031727100. 12740444 PMC164495

[36] Marquis JF, LaCourse R, Ryan L, North RJ, Gros P 2009 Genetic and functional characterization of the mouse Trl3 locus in defense against tuberculosis. J Immunol 182:3757–3767. doi:10.4049/jimmunol.0802094. 19265154 PMC4301439

[37] Svenson KL, Gatti DM, Valdar W, Welsh CE, Cheng R, Chesler EJ, Palmer AA, McMillan L, Churchill GA 2012 High-resolution genetic mapping using the mouse Diversity Outbred population. Genetics 190:437–447. doi:10.1534/genetics.111.132597. 22345611 PMC3276626

[38] Niazi MKK, Dhulekar N, Schmidt D, Major S, Cooper R, Abeijon C, Gatti DM, Kramnik I, Yener B, Gurcan M, Beamer G 2015 Lung necrosis and neutrophils reflect common pathways of susceptibility to Mycobacterium tuberculosis in genetically diverse, immune-competent mice. Dis Model Mech 8:1141–1153. doi:10.1242/dmm.020867. 26204894 PMC4582107

[39] Churchill GA, Airey DC, Allayee H, Angel JM, Attie AD, Beatty J, Beavis WD, Belknap JK, Bennett B, Berrettini W, Bleich A, Bogue M, Broman KW, Buck KJ, Buckler E, Burmeister M, Chesler EJ, Cheverud JM, Clapcote S, Cook MN, Cox RD, Crabbe JC, Crusio WE, Darvasi A, Deschepper CF, Doerge RW, Farber CR, Forejt J, Gaile D, Garlow SJ, Geiger H, Gershenfeld H, Gordon T, Gu J, Gu W, de Haan G, Hayes NL, Heller C, Himmelbauer H, Hitzemann R, Hunter K, Hsu H-C, Iraqi FA, Ivandic B, Jacob HJ, Jansen RC, Jepsen KJ, Johnson DK, Johnson TE, Kempermann G, 2004 The Collaborative Cross, a community resource for the genetic analysis of complex traits. Nat Genet 36:1133–1137. doi:10.1038/ng1104-1133. 15514660

[40] Collaborative Cross Consortium. 2012 The genome architecture of the Collaborative Cross mouse genetic reference population. Genetics 190:389–401. doi:10.1534/genetics.111.132639. 22345608 PMC3276630

[41] Smith CM, Proulx MK, Olive AJ, Laddy D, Mishra BB, Moss C, Gutierrez NM, Bellerose MM, Barreira-Silva P, Phuah JY, Baker RE, Behar SM, Kornfeld H, Evans TG, Beamer G, Sassetti CM 2016 Tuberculosis susceptibility and vaccine protection are independently controlled by host genotype. mBio 7:e01516-16. doi:10.1128/mBio.01516-16. PMC5030360 27651361

[42] Sakai S, Kauffman KD, Sallin MA, Sharpe AH, Young HA, Ganusov VV, Barber DL 2016 CD4 T cell-derived IFN-γ plays a minimal role in control of pulmonary Mycobacterium tuberculosis infection and must be actively repressed by PD-1 to prevent lethal disease. PLoS Pathog 12:e1005667. doi:10.1371/journal.ppat.1005667. 27244558 PMC4887085

[43] Shorter JR, Najarian ML, Bell TA, Blanchard M, Ferris MT, Hock P, Kashfeen A, Kirchoff KE, Linnertz CL, Sigmon JS, Miller DR, McMillan L, Pardo-Manuel de Villena F 2019 Whole genome sequencing and progress toward full inbreeding of the mouse Collaborative Cross population. G3 (Bethesda) 9:1303–1311. doi:10.1534/g3.119.400039. 30858237 PMC6505143

[44] Aylor DL, Valdar W, Foulds-Mathes W, Buus RJ, Verdugo RA, Baric RS, Ferris MT, Frelinger JA, Heise M, Frieman MB, Gralinski LE, Bell TA, Didion JD, Hua K, Nehrenberg DL, Powell CL, Steigerwalt J, Xie Y, Kelada SNP, Collins FS, Yang IV, Schwartz DA, Branstetter LA, Chesler EJ, Miller DR, Spence J, Liu EY, McMillan L, Sarkar A, Wang J, Wang W, Zhang Q, Broman KW, Korstanje R, Durrant C, Mott R, Iraqi FA, Pomp D, Threadgill D, de Villena F-M, Churchill GA 2011 Genetic analysis of complex traits in the emerging Collaborative Cross. Genome Res 21:1213–1222. doi:10.1101/gr.111310.110. 21406540 PMC3149489

[45] Earl PL, Americo JL, Moss B 2012 Lethal monkeypox virus infection of CAST/EiJ mice is associated with a deficient gamma interferon response. J Virol 86:9105–9112. doi:10.1128/JVI.00162-12. 22696658 PMC3416162

[46] Earl PL, Americo JL, Moss B 2017 Insufficient innate immunity contributes to the susceptibility of the castaneous mouse to orthopoxvirus infection. J Virol 91:e01042-17. doi:10.1128/JVI.01042-17. 28747505 PMC5599762

[47] Baaten BJG, Li C-R, Deiro MF, Lin MM, Linton PJ, Bradley LM 2010 CD44 regulates survival and memory development in Th1 cells. Immunity 32:104–115. doi:10.1016/j.immuni.2009.10.011. 20079666 PMC2858628

[48] Seder RA, Darrah PA, Roederer M 2008 T-cell quality in memory and protection: implications for vaccine design. Nat Rev Immunol 8:247–258. doi:10.1038/nri2274. 18323851

[49] Srivastava A, Morgan AP, Najarian ML, Sarsani VK, Sigmon JS, Shorter JR, Kashfeen A, McMullan RC, Williams LH, Giusti-Rodríguez P, Ferris MT, Sullivan P, Hock P, Miller DR, Bell TA, McMillan L, Churchill GA, de Villena F-M 2017 Genomes of the mouse Collaborative Cross. Genetics 206:537–556. doi:10.1534/genetics.116.198838. 28592495 PMC5499171

[50] Szafranski K, Schindler S, Taudien S, Hiller M, Huse K, Jahn N, Schreiber S, Backofen R, Platzer M 2007 Violating the splicing rules: TG dinucleotides function as alternative 3′ splice sites in U2-dependent introns. Genome Biol 8:R154. doi:10.1186/gb-2007-8-8-r154. 17672918 PMC2374985

[51] Zhang J, Teh M, Kim J, Eva MM, Cayrol R, Meade R, Nijnik A, Montagutelli X, Malo D, Jaubert J 2019 A loss-of-function mutation in *Itgal* contributes to the high susceptibility of Collaborative Cross strain CC042 to *Salmonella* infections. bioRxiv doi:10.1101/723478. PMC6921657 31636138

[52] Ardain A, Domingo-Gonzalez R, Das S, Kazer SW, Howard NC, Singh A, Ahmed M, Nhamoyebonde S, Rangel-Moreno J, Ogongo P, Lu L, Ramsuran D, de la Luz Garcia-Hernandez M, Ulland TK, Darby M, Park E, Karim F, Melocchi L, Madansein R, Dullabh KJ, Dunlap M, Marin-Agudelo N, Ebihara T, Ndung’u T, Kaushal D, Pym AS, Kolls JK, Steyn A, Zúñiga J, Horsnell W, Yokoyama WM, Shalek AK, Kløverpris HN, Colonna M, Leslie A, Khader SA 2019 Group 3 innate lymphoid cells mediate early protective immunity against tuberculosis. Nature 570:528–532. doi:10.1038/s41586-019-1276-2. 31168092 PMC6626542

[53] Treerat P, Prince O, Cruz-Lagunas A, Muñoz-Torrico M, Salazar-Lezama MA, Selman M, Fallert-Junecko B, Reinhardt TA, Alcorn JF, Kaushal D, Zuñiga J, Rangel-Moreno J, Kolls JK, Khader SA 2017 Novel role for IL-22 in protection during chronic Mycobacterium tuberculosis HN878 infection. Mucosal Immunol 10:1069–1081. doi:10.1038/mi.2017.15. 28247861 PMC5477058

[54] Sallin MA, Kauffman KD, Riou C, Du Bruyn E, Foreman TW, Sakai S, Hoft SG, Myers TG, Gardina PJ, Sher A, Moore R, Wilder-Kofie T, Moore IN, Sette A, Lindestam Arlehamn CS, Wilkinson RJ, Barber DL 2018 Host resistance to pulmonary Mycobacterium tuberculosis infection requires CD153 expression. Nat Microbiol 3:1198–1205. doi:10.1038/s41564-018-0231-6. 30202016

[55] Threadgill DW, Churchill GA 2012 Ten years of the Collaborative Cross. Genetics 190:291–294. doi:10.1534/genetics.111.138032. 22345604 PMC3276648

[56] Welsh CE, Miller DR, Manly KF, Wang J, McMillan L, Morahan G, Mott R, Iraqi FA, Threadgill DW, de Villena F-M 2012 Status and access to the Collaborative Cross population. Mamm Genome 23:706–712. doi:10.1007/s00335-012-9410-6. 22847377 PMC3463789

