# Supplementary material for: Functionally Overlapping Variants Control Tuberculosis Susceptibility in Collaborative Cross Mice
Source: mBio. 2019 Nov 26;10(6):e02791-19. doi: 10.1128/mBio.02791-19 (PMC6879725; doi:10.1128/mBio.02791-19)
Supplement: FIG S1 [file mBio.02791-19-sf001.pdf]

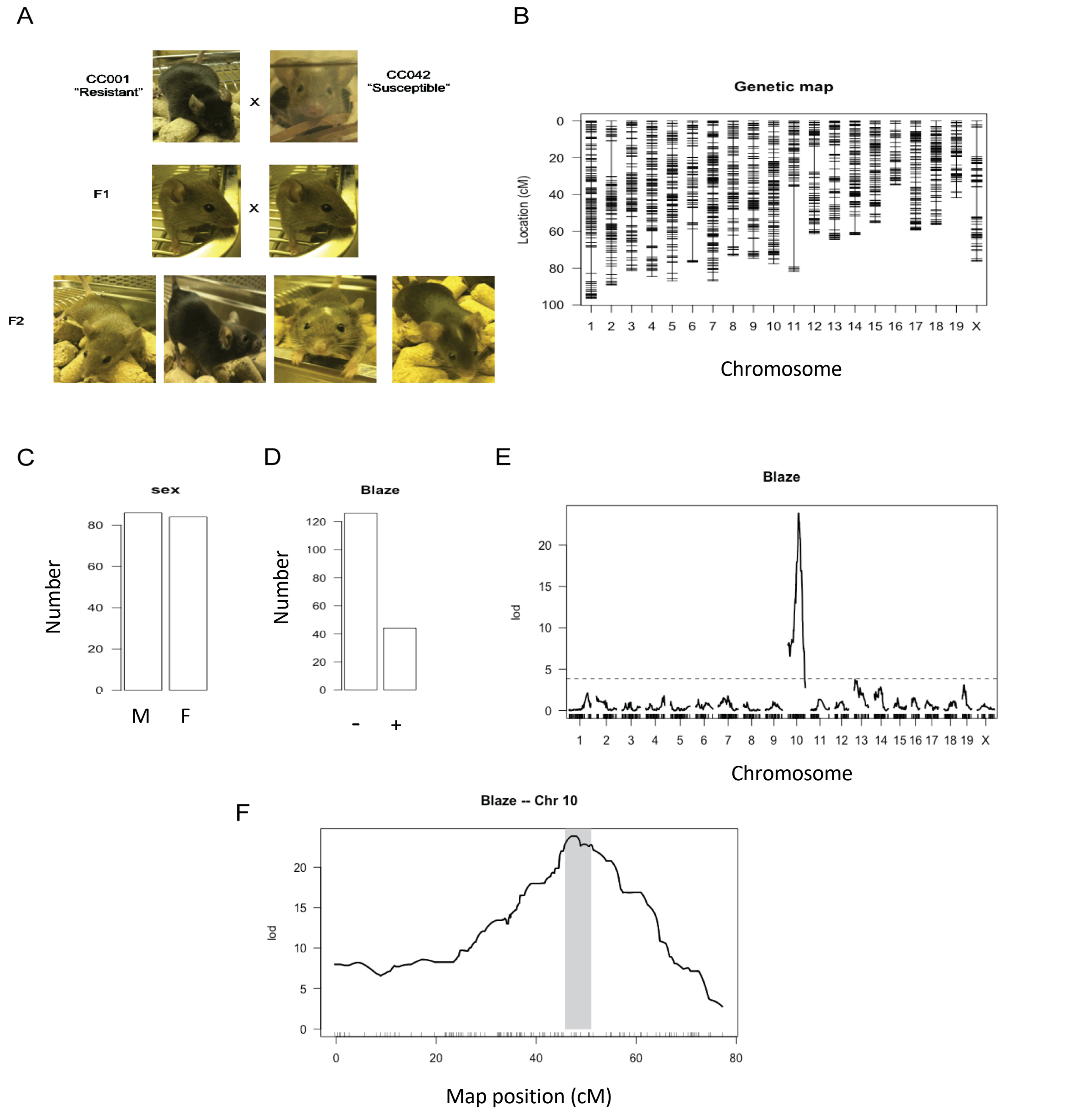

Supplemental Figure 1. Description of F2 cross population and test scan for the Mendelian "Blaze" trait. (A) Coat color phenotypes of parent mice, CC001 (black), CC042 (agouti with white head blaze), F_1 (agouti) and F_2 (agouti, black, agouti and head blaze, black and head blaze). (B) genetic map of F2 population used in QTL studies. Vertical lines show Chromosomes (1-19 autosomes, X = X Chromosome). Horizontal ticks show marker location in cM. (C) Number of male vs. female mice (male=M, female=F). (D) Number of mice with no head blaze vs head blaze in F2 mice (no head blaze= - ; head blaze= +). (E) Results of genome scan for "Blaze" trait. (F) Bayes interval for the "Blaze" trait on chromosome 10 (shaded) containing *Kitl*, previously shown to be associated with the WSB^{blaze} phenotype (Aylor DL *et al.* 2011. Genome Research 21:1213–1222).
