# Supplementary material for: Functionally Overlapping Variants Control Tuberculosis Susceptibility in Collaborative Cross Mice
Source: mBio. 2019 Nov 26;10(6):e02791-19. doi: 10.1128/mBio.02791-19 (PMC6879725; doi:10.1128/mBio.02791-19)
Supplement: FIG S2 [file mBio.02791-19-sf002.pdf]

A

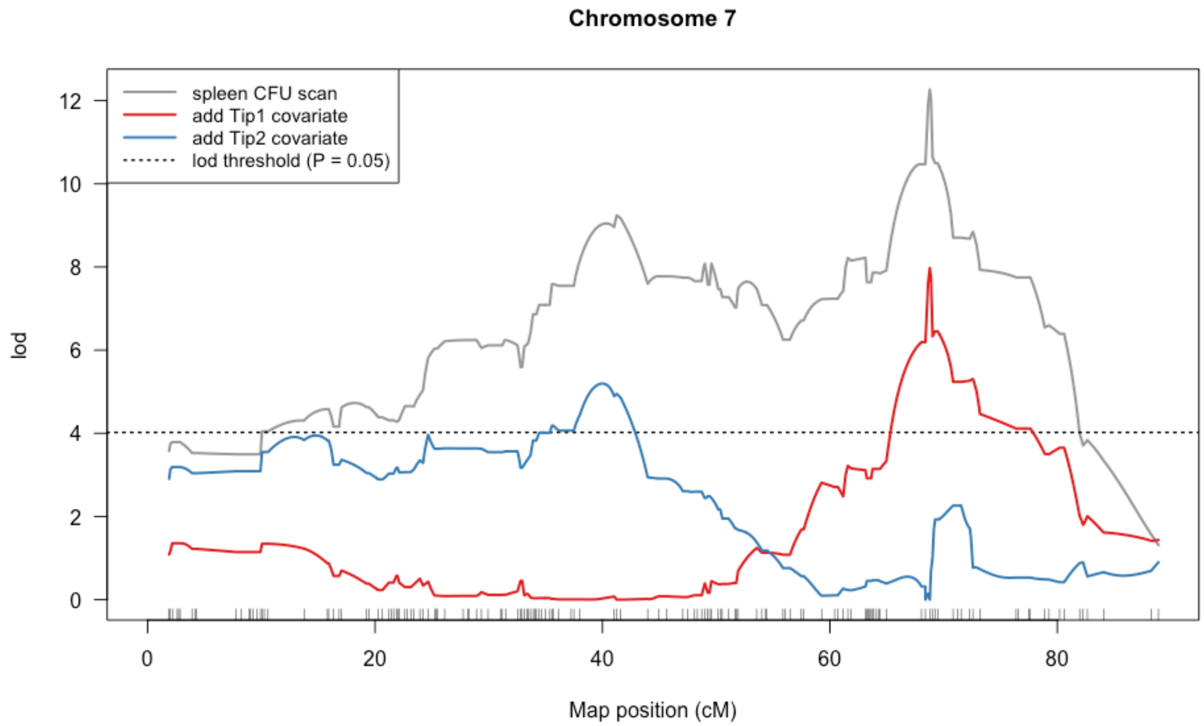

B

	df	Type III SS	LOD	%var	F value	Pvalue(Chi2)	Pvalue(F)
Tip1	2	5.78	4.99	6.79	11.52	1.01E-05	2.14E-05
Tip2	2	10.37	8.53	12.19	20.67	2.95E-09	1.06E-08
Tip3	2	2.55	2.29	2.99	5.08	5.18E-03	7.30E-03

Supplemental Figure 2. Tests for independence of Tip1 and Tip2 QTL underlying spleen CFU. (A) The spleen CFU trait was re-mapped using the genotype probabilities at Tip1 and Tip2 separately as covariates. The distal QTL (Tip2) QTL reaches significant LOD after variation explained by Tip1 was removed and vice versa. (B) A multi-QTL model including Tip1, Tip2, and Tip3 was fit for the spleen CFU phenotype (batch and sex included as covariates). The table shows results from drop-one-term ANOVA where each QTL is dropped from the model, one at a time, and the sub-model with that factor omitted is compared to the full model. The results provide substantial evidence for all QTL. Column headings: df, degrees of freedom; Type III SS, Type III sum of squares; LOD, LOD score; %var, percentage of variance explained; Pvalue(Chi2), P value for chi square; Pvalue(F), P value for F distribution. The degree of freedom, Type III sum of squares, LOD score, and percentage of variance explained are the values comparing the full to the sub-model.
