# Supplementary material for: Functionally Overlapping Variants Control Tuberculosis Susceptibility in Collaborative Cross Mice
Source: mBio. 2019 Nov 26;10(6):e02791-19. doi: 10.1128/mBio.02791-19 (PMC6879725; doi:10.1128/mBio.02791-19)
Supplement: FIG S3 [file mBio.02791-19-sf003.pdf]

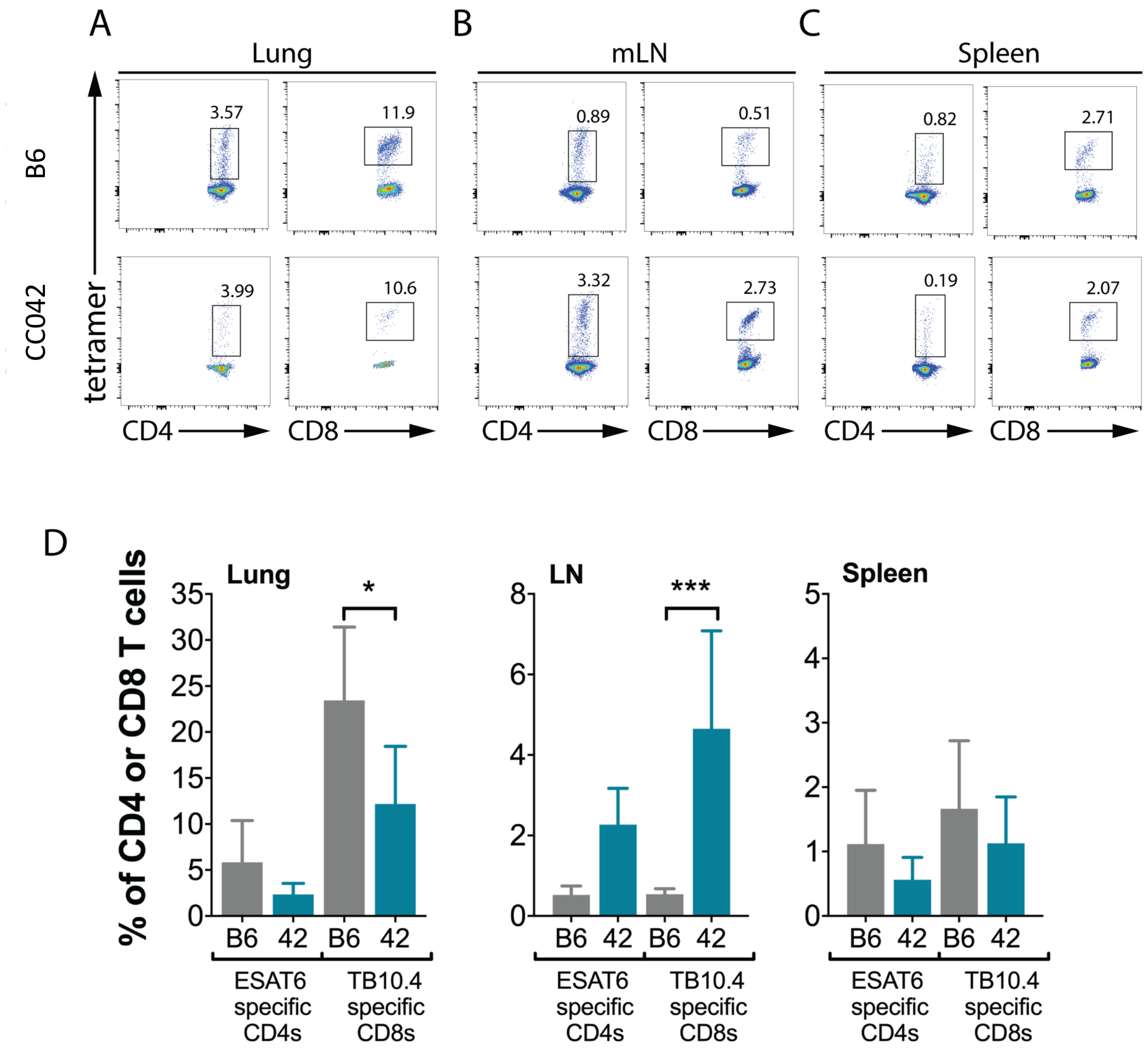

Supplemental Figure 3. Frequency of antigen-specific T cells in B6 and CC042 following pulmonary Mtb infection. Representative flow plots showing the frequencies of ESAT-6-specific CD4 T cells and TB10.4-specific CD8 T cells in the (A) Lung (B) mediastinal lymph node (mLN) and (C) spleen of B6 (grey shading) and CC042 (teal shading) mice at 4 weeks post pulmonary Mtb infection. Bar plots show mean + SD of ESAT-6-specific CD4 T cells and TB10.4-specific CD8 T cells in the (D) Lung (E) mLN and (F) spleen at the same timepoint. Sidak's multiple comparison test was used to determine significance where $p<0.05$ *, $p<0.001$***.
